# Accelerated Extracellular Nucleotide Metabolism in Brain Microvascular Endothelial Cells in Experimental Hypercholesterolemia

**DOI:** 10.1007/s10571-023-01415-8

**Published:** 2023-10-06

**Authors:** Ewelina Czuba-Pakuła, Iwona Pelikant-Małecka, Grażyna Lietzau, Sławomir Wójcik, Ryszard T. Smoleński, Przemysław Kowiański

**Affiliations:** 1 Division of Anatomy and Neurobiology, Faculty of Medicine, Medical University of Gdańsk, Dębinki 1, 80-211 Gdańsk, Poland; 2 Division of Medical Laboratory Diagnostics - Fahrenheit Biobank BBMRI.pl, Medical University of Gdańsk, Dębinki 1, 80-211 Gdańsk, Poland; 3 Department of Biochemistry, Faculty of Medicine, Medical University of Gdańsk, Dębinki 1, 80-211 Gdańsk, Poland; 4 Institute of Health Sciences, Pomeranian University in Słupsk, Bohaterów Westerplatte 64, 76-200 Słupsk, Poland

**Keywords:** Ecto-enzymes, Endothelium, Glycolysis, Hypercholesterolemia, Purinergic nucleotides, Stroke

## Abstract

Hypercholesterolemia affects the neurovascular unit, including the cerebral blood vessel endothelium. Operation of this system, especially in the context of energy metabolism, is controlled by extracellular concentration of purines, regulated by ecto-enzymes, such as e-NTPDase-1/CD39, ecto-5′-NT/CD73, and eADA. We hypothesize that hypercholesterolemia, via modulation of the activity of nucleotide metabolism-regulating ecto-enzymes, deteriorates glycolytic efficiency and energy metabolism of endothelial cells, which may potentially contribute to development of neurodegenerative processes. We aimed to determine the effect of hypercholesterolemia on the concentration of purine nucleotides, glycolytic activity, and activity of ecto-enzymes in the murine brain microvascular endothelial cells (mBMECs). We used 3-month-old male LDLR^−/−^/Apo E^−/−^ double knockout mice to model hypercholesterolemia and atherosclerosis. The age-matched wild-type C57/BL6 mice were a control group. The intracellular concentration of ATP and NAD and extracellular activity of the ecto-enzymes were measured by HPLC. The glycolytic function of mBMECs was assessed by means of the extracellular acidification rate (ECAR) using the glycolysis stress test. The results showed an increased activity of ecto-5′-NT and eADA in mBMECs of the hypercholesterolemic mice, but no differences in intracellular concentration of ATP, NAD, and ECAR between the hypercholesterolemic and control groups. The changed activity of ecto-5′-NT and eADA leads to increased purine nucleotides turnover and a shift in their concentration balance towards adenosine and inosine in the extracellular space. However, no changes in the energetic metabolism of the mBMECs are reported. Our results confirm the influence of hypercholesterolemia on regulation of purine nucleotides metabolism, which may impair the function of the cerebral vascular endothelium.

**Graphical abstract:**

The effect of hypercholesterolemia on the murine brain microvascular endothelial cells (mBMECs). An increased activity of ecto-5′-NT and eADA in mBMECs of the LDLR^−/−^/Apo E^−/−^ mice leads to a shift in the concentration balance towards adenosine and inosine in the extracellular space with no differences in intracellular concentration of ATP. Figure was created with Biorender.com.

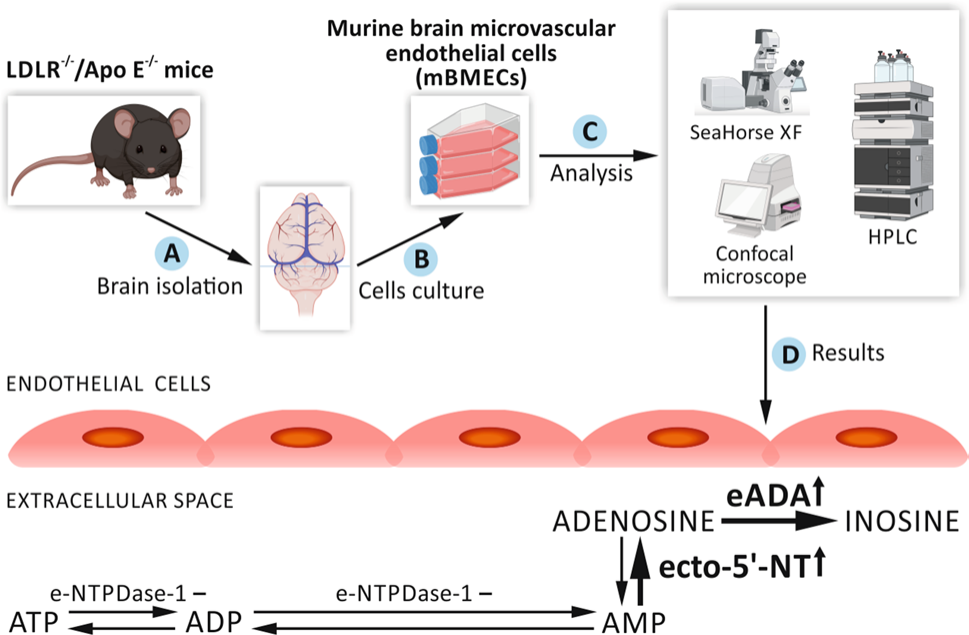

## Introduction

Hypercholesterolemia is a risk factor for numerous metabolic and neurodegenerative diseases, such as diabetes, arterial hypertension, and Alzheimer’s disease (Ghribi et al. 2006a; Gosselet et al. 2014; Chen et al. 2016; Czuba et al. 2017). Despite the increasing use of the lipid-lowering drugs (Saeed et al. 2015), this lipid disorder is still regarded as one of the major risk factors for ischemic stroke (Engström et al. 2002; Macrez et al. 2011). At the base of these pathologies are hypercholesterolemia-induced energetic metabolism impairment, cerebral blood flow dysregulation, leakage of the blood–brain barrier (BBB), and activation of the inflammatory response (Rajeev et al. 2022; Czuba-Pakuła et al. 2023). The damage of BBB has also been reported in the course of neurodegenerative diseases (Gosselet et al. 2014; Sweeney et al. 2019; De Oliveira et al. 2020). In all these processes, the role of the purinergic system is postulated, although the evidence of hypercholesterolemia influence is a subject of continuous studies. The purinergic signaling system relays on multidirectional interactions, which enables the functional integration of glial cells, neurons, and brain vascular endothelial cells (Fields and Burnstock 2006). In pathological conditions, released from damaged cells purines act as signaling molecules that affect intercellular interactions and induce the release of cytokines and expression of adhesion molecules (Giaume et al. 2007). Purine nucleotides modulate functions of the nervous tissue and vascular system, as well as inflammatory reactions initiated by the external and internal stimuli (Bours et al. 2006; Kutryb-Zajac et al. 2018a, c).

The majority of pathological factors responsible for damage of the nervous tissue lead to the release of metabolites, neurotransmitters, and signaling molecules which are responsible for the initiation of destructive processes like inflammatory response, oxidative stress, anaerobic glycolysis followed by lactacidosis and, finally, cellular death both necrotic and apoptotic. The character and intensity of all these processes vary considerably depending on the nature of a particular stimulus, its intensity, duration of the exposure, brain area, as well as animal age and species. Hypercholesterolemia-induced changes that affect endothelial cell function include leakage of BBB, activation of the inflammatory mediators, energy metabolism impairment, and cerebral blood flow dysregulation [reviewed in Czuba et al. (2017)].

Taking into account the complex nature of the nervous tissue structure and the functional relationships of its morphological elements (reflected in the concepts of the neurovascular unit and neurovascular coupling), it can be expected that the effects of pathological stimuli will be different in various cell populations present in the brain. Hence, the changes occurring in the endothelium of brain vessels in the course of hypercholesterolemia should be seen from this perspective. It can be assumed that the hypercholesterolemia studied in our experimental model is not a strong destructive stimulus, which leads to damage to the brain tissue and its vascular system in a short time. Consequently, the changes in the energy metabolism may be limited or may not occur at all.

Previous research focused rather on metabolic dysfunction occurring in the endothelial cells of peripheral vessels than in the brain endothelium (reviewed in publications by Kutryb-Zając et al. cited in our manuscript). Overall, this is an important argument that underlines the value of our work, which fills a gap of knowledge in this area.

Adenosine triphosphate (ATP) is the key element of energy metabolism, which while outside the cell, also acts as a neurotransmitter affecting the activity of neurons, astrocytes, and microglia (Boué-Grabot and Pankratov 2017). Acting via purinergic receptors, it shapes the nervous tissue response to different types of damage (Bjelobaba et al. 2011). ATP induces a pro-inflammatory effect, contributing to the release of cytokines, e.g., IL-1β, IL-2, IFN-γ, and TNF-α from neuroglia (Polachini et al. 2014). ATP also plays an important role as a neuro- and gliotransmitter, spreading in waves to maintain Ca^2+^-dependent signaling (Bezzi and Volterra 2001). The role of ATP in the mechanism of calcium wave propagation has been reported in various regions of the brain, suggesting the presence of an alternative signaling pathway (Wang et al. 2000; Bezzi and Volterra 2001). Therefore, small changes in purinergic signaling between glial cells and neurons can have profound effects leading to the development of pathological processes. Whereas under physiological conditions, the extracellular concentration of ATP is in a very low nanomolar range, in pathological processes, its concentration increases in the extracellular space as a result of its release from damaged cells (Pearson et al. 2003; Fields and Burnstock 2006). In many cases, ATP reveals an inhibitory effect on the cellular signal transmission (Roszek and Czarnecka 2015). Other components of the purinergic system exert different effects on brain tissue. Previous studies have shown that adenosine has potent anti-inflammatory and immunosuppressive effects, while inosine plays immunomodulatory and neuroprotective functions in the brain (Muto et al. 2014; Polachini et al. 2014).

**Fig. 1:**
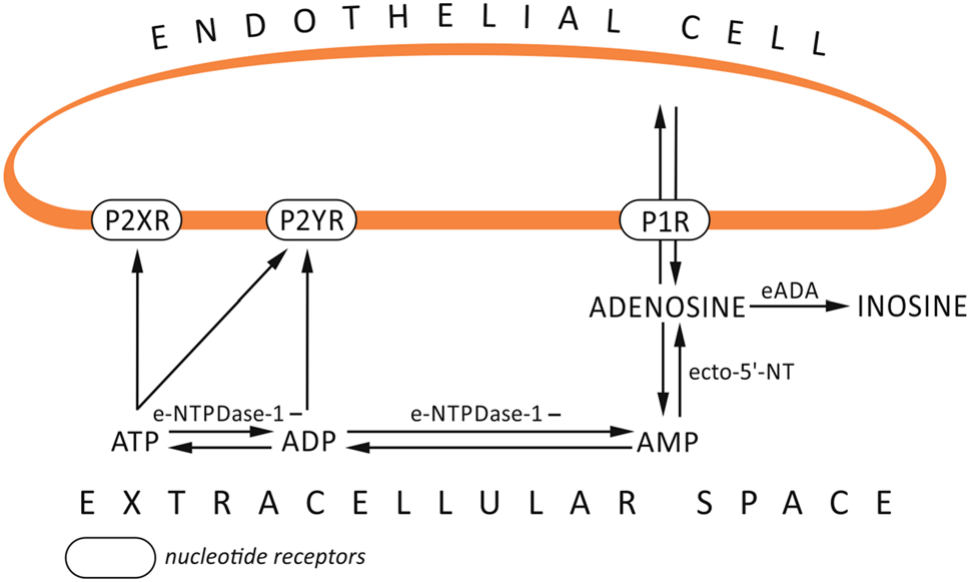
The role of purine nucleotides turnover enzymes in ATP metabolism. In the endothelial cell, purines are metabolized by ecto-enzymes anchored to the plasma membrane with their active sites situated outside the cell. The ecto-nucleoside triphosphate diphosphohydrolase-1 (e-NTPDase-1) hydrolyzes ATP to ADP and AMP, which is then converted by ecto-5′-nucleotidase (ecto-5′-NT) to adenosine and by ecto-adenosine deaminase (eADA) to inosine. Both adenosine and adenine nucleotides are transported bi-directionally between the cytoplasm and extracellular space through nucleoside transporters. P2XR - P2X purinoreceptors, P2YR - P2Y purnoreceptors, P1R - P1 purinoreceptors

In the extracellular space, ATP is metabolized by ecto-enzymes, anchored to the plasma membrane, with their active sites situated outside the cell. One of the ecto-enzymes is ecto-nucleoside triphosphate diphosphohydrolase-1 (e-NTPDase-1/CD39) that hydrolyzes ATP and ADP to AMP (Chang et al. 2021) (Fig. 1).

The latter is hydrolyzed by ecto-5′-nucleotidase (ecto-5′-NT/CD73) to adenosine and by ecto-adenosine deaminase (eADA) to inosine (Polachini et al. 2014). Apart from the above-mentioned metabolic pathway, the extracellular ATP may be converted to deoxyadenosine monophosphate (DAMP), which induces a reactive astrocyte response, leading to the increased release of inflammatory cytokines (Brisevac et al. 2013). The presence of e-NTPDase-1 in microglia, cerebral vascular endothelium, and smooth muscles suggests its role in the regulation of several physiological and pathological processes (Braun et al. 2000). Although e-NTPDase-1 is not present in neurons and astrocytes, it can indirectly regulate their functions by hydrolyzing purine nucleotides in the extracellular space (Braun et al. 2000; Harter et al. 2018). The other ecto-enzyme, ecto-5′-NT, is an important regulator of the inflammatory process in the central nervous system (CNS) (Brisevac et al. 2012a). By hydrolyzing AMP to adenosine, ecto-5′-NT changes the dynamic balance, reducing the proportion between the pro-inflammatory factor (i.e., ATP) and the anti-inflammatory, immunosuppressive one (i.e., adenosine). Increased expression of this enzyme has been reported in several in vivo models of brain injury (Brisevac et al. 2012b). Another ecto-enzyme, eADA, has a neuroprotective function in several brain areas in rats (Tamura et al. 2016). The results of the previous study have suggested that modulation of eADA activity may prove to be an effective therapeutic strategy to limit the extent of brain damage in patients with atheromathosis (Dirnagl et al. 1999).

The dynamics of metabolic transformations of purines in the brain extracellular space and changes in energy metabolism in the brain microvascular endothelial cells (mBMECs) in hypercholesterolemia have not been fully elucidated. Hence, in this study, we aimed to determine the effect of hypercholesterolemia on the activity of the ecto-enzymes critical for the purine metabolism in the CNS. We also aimed to evaluate the hypercholesterolemia-induced alterations in ATP and NAD levels in the mBMECs, and changes in the energetic metabolism based on glycolytic function: glycolytic capacity, glycolytic reserve, and non-glycolytic acidification in mBMECs of LDLR^−/−^/Apo E^−/−^ double knockout mice versus the control mice.

## Materials and Methods

### Animal Model and Study Design

The mice were obtained from the Department of Biochemistry and Tri-City Academic Laboratory Animal Centre—Research and Services Centre at the Medical University of Gdansk. Twelve 3-month-old male LDLR^−/−^/Apo E^−/−^ double knockout mice were used as in vivo models of hypercholesterolemia and atherosclerosis (Zadelaar et al. 2007; Gajda et al. 2008; Getz and Reardon 2016; Kutryb-Zajac et al. 2018b). The LDLR^−/−^/Apo E^−/−^ double knockout mouse model has previously been used as a validated and sufficient model of hypercholesterolemia and atherosclerosis in vitro. This is dictated by the fact that several tests have been performed on this model, which has been documented in publications by other authors (Zadelaar et al. 2007; Gajda et al. 2008; Kutryb-Zajac et al. 2018b). In this model, parameters such as total plasma cholesterol, LDL cholesterol, HDL cholesterol, and triglycerides were checked. Additionally, fasting blood glucose concentration and blood cell count parameters were studied. The results have shown that LDLR^−/−^/Apo E^−/−^ mice developed spontaneous atherosclerosis faster than ApoE^−/−^ mice and they did not require a diet manipulation like LDLR^−/−^ mice. Thus, the study showed that the LDLR^−/−^/Apo E^−/−^ double knockout genetic model is the most accurate and validated in vitro model of hypercholesterolemia in humans. In humans, this mutation is associated with a dyslipidemic pattern that includes familial hypercholesterolemia, type III hyperlipidemia, and increased susceptibility to atherosclerosis (Breslow 1996; Mahley and Bersot 2006). The control group consisted of 12 age-matched wild-type male C57/BL6 mice. Animals were housed in individually ventilated cages, not exceeding 5 individuals per cage, at 22.5 ± 0.5 °C, 40 ± 5% humidity, in 12-h alternating day/night cycles with unlimited access to tap water and standard chow.

This project was accepted by the Local Ethical Committee of the Medical University of Gdansk (WHiBZ/lke.003/29/18). All animal handling procedures and experimental protocols were performed under the provisions of the EU Council Directive 2010/63/EU for animal experiments, with the preservation of humanitarian care and the use of laboratory animals to minimize animals’ pain and discomfort and to reduce the number of experimental subjects. The study is reported according to the ARRIVE guidelines (Percie du Sert et al. 2020).

The authors were blinded to the experimental protocol while performing the experiments and the statistical calculations.

### Isolation and Identification of Murine Brain Microvascular Endothelial Cells

Murine brain microvascular endothelial cells (mBMECs) were isolated from the brains of the wild-type C57/BL6 mice and LDLR^−/−^/Apo E^−/−^ double knockout mice. Mice were anesthetized with a lethal dose of ketamine (100 mg/kg) and xylazine (10 mg/kg) that was injected intraperitoneally and then decapitated. Murine brains were removed from the skulls in the laminar chamber under sterile conditions, cut into small pieces, and placed in Dulbecco’s Modified Eagle’s Medium (DMEM; SIGMA, UK) with low glucose, supplemented with 10% Fetal Bovine Serum (FBS; SIGMA, UK), 2 mM glutamine, 1 mM sodium pyruvate, and penicillin–streptomycin. The tissue was centrifuged twice, and the erythrocytes found above the precipitate were removed. The pellet was suspended in 0.1% Collagenase Type A solution (1 mg/mL in DMEM low glucose; ROCHE, UK) and incubated with shaking at 37 °C for 1 h. The single-cell suspension was passed through a 70 µm strainer into DMEM, centrifuged, and suspended in BSA-DMEM solution (20% w/v). After another centrifugation upper myelin layer was removed. Then, the pellets were transferred to a culture flask (25 cm^2^) and plated in DMEM with d-Valine (without l-Valine) (glucose 4.5 g/L; SIGMA, UK), supplemented with 10% FBS (SIGMA, UK), 2 mM glutamine, and penicillin–streptomycin. d-valine suppresses the growth of astrocytes (Freyer et al. 1999) and allows to remove fibroblasts from co-culture after isolation (Akis and Madaio 2004). After all performed manipulations mostly endothelial cells remain (about 90–95% of the population are endothelial cells).

### Murine Brain Microvascular Endothelial Cells (mBMECs) Culture

mBMECs were grown in DMEM with d-Valine (without l-Valine) (glucose 4.5 g/L; SIGMA, UK), supplemented with 10% FBS (SIGMA, UK), endothelial cell growth supplement (ECGS; SIGMA, UK), 2 mM glutamine, and penicillin–streptomycin at 37 °C in an atmosphere of 5% CO_2_. The wild-type C57/BL6 mice and LDLR^−/−^/Apo E^−/−^ double knockout mice cell cultures were separated from each other in culture flasks, and no cells were pooled. After reaching 70–90% confluent by cells, they were washed with BSA solution and harvested with trypsin.

### Determination of Nucleotide Concentrations

For further procedures, the wild-type C57/BL6 mice and LDLR^−/−^/Apo E^−/−^ double knockout mice cells were plated on a 24-well plate at a density of 10 × 10^4^ per well. After 24 h, the medium was changed, and cells were growing until 90% of confluence. The next step of the procedure required separating the cells from the medium, all medium was removed, and cells were washed gently with Hanks’ Balanced Salt Solution two times (HBSS; SIGMA, UK). For extraction of the cellular ATP and NAD, 0.3 mL of 0.4 M HClO_4_ was added to each well, and then plates were frozen at − 80 °C. The cell extracts were thawed on ice, collected and centrifuged (14000 rpm, 5 min) to remove protein precipitate. The supernatants were then adjusted to pH 5.5–6.0 using 3 M K_3_PO_4_. After 15-min incubation on ice and centrifugation (14000 rpm, 5 min), supernatants were analyzed by HPLC (Smolenski et al. 2006). Protein precipitates were dissolved in 0.5 mL 0.5 M NaOH and analyzed with the Bradford method (Bradford 1976) with the use of bovine albumin as a calibration standard.

### Glycolytic Function Analysis

Glycolytic function in mBMECs was measured by means of the Seahorse XFp Glycolysis Stress Test kit (Agilent, USA) using a Seahorse XFp metabolic flux analyzer (Agilent), following the manufacturer’s instructions. Cells were plated on 8-well Seahorse XFp Microplates, according to the manufacturer’s protocol, to reach 90% of confluence in a final volume of 200 µL. After overnight incubation, the cells were washed gently twice with Seahorse XF Base Medium supplemented with 2 mM glutamine (pH 7.4). Each well was supplemented with 180 µL of warm Seahorse XF Base Medium with glutamine and incubated at 37 °C for 45 min without CO_2_. The Glycolysis Stress Test was performed with serial injections of glucose, oligomycin (an ATP synthase inhibitor), and 2-deoxy-glucose (2-DG; a hexokinase inhibitor) at final concentrations of 10 mM, 1.5 μM, and 50 mM. The measurement of the extracellular acidification rate (ECAR) at each step, which is a consequence of the net production and extrusion of protons into the extracellular medium, allowed for determining Glycolytic Capacity and Glycolysis Rate. Both of these parameters were necessary to calculate the Glycolytic Reserve.

Glycolysis was determined as the ECAR rate reached by the cells upon saturation with glucose. The glycolytic capacity indicates the maximum ECAR rate reached by the cell being forced to use glycolysis to its maximum capacity. The glycolytic reserve indicates the capability of a cell to respond to an energetic demand. In contrast, non-glycolytic acidification measures other sources of extracellular acidification that are not attributed to glycolysis. The ECAR values obtained were normalized to the protein concentration, which was measured using the Bradford method (Bradford 1976). All experiments were carried out three times in constant conditions.

### The Extracellular Activity of Ecto-enzymes: Ecto-nucleoside Triphosphate Diphosphohydrolase-1, ecto-5′-nucleotidase, and Adenosine Deaminase

The extracellular activity of ecto-nucleoside triphosphate diphosphohydrolase-1 (e-NTPDase-1/CD39), ecto-5′-nucleotidase (ecto-5′-NT/CD37), and adenosine deaminase (eADA) was measured on 24-well plates. For the experiments, cells were plated at a density of 10 × 10^4^ per well. After 24 h, the medium was changed and cells were grown until 90% of confluence. Then, the cells were washed gently twice with HBSS (SIGMA, UK), supplemented with 25 mM N-2-hydroxyethylpiperazine-N-2-ethane sulfonic acid (HEPES) and 1 g/L glucose. Subsequently, the cells were incubated for 15 min in the supplemented HBSS solution with erythro-9-(2-hydroxy-3-nonyl)adenine (EHNA; SIGMA, UK), in final concentration 5 µM (EHNA; SIGMA, UK). After this time, appropriate substrates such as ATP, AMP, and adenosine were added to the cells at a concentration of 50 µM, and samples were collected at time points after 0, 5, 15, and 30 min. All reactions were stopped by freezing 24-well plates at a temperature of  −  80 °C for 24 h. Extracellular activities of e-NTPDase-1, ecto-5′-NT, and eADA enzymes were analyzed as a conversion of extracellular nucleotides into their products by high-performance liquid chromatography (HPLC) (Smolenski et al. 2006). All experiments were performed three times in constant conditions.

### Tissue Preparation for Immunohistochemistry

Mice were intraperitoneally injected with a lethal dose of ketamine (100 mg/kg) and xylazine (10 mg/kg). Next, the arterial cannula was introduced into the arch of the aorta through the apex of the heart, and the mice were perfused transcardially with physiological saline solution (NaCl 0.9%; pH 7.4; 150 mL) and 4% paraformaldehyde in phosphate-buffered saline (0.1 M; pH 7.4; 150 mL; PBS; Sigma, UK) at RT. Mouse brains were extracted from the skull and postfixed in 4% paraformaldehyde solution for 2 h at 4 °C. The brains were then cryoprotected by immersion in 15% and 30% sucrose solutions in 0.1 M PBS (pH 7.4; 4 °C; Sigma, UK) until sinking. Subsequently, they were frozen at − 20 °C, and cut into 40-µm-thick sections on a freezing cryostat (Thermo Scientific Cryostat Microm HM 525, Germany).

### Immunohistochemistry

The localization of the e-NTPDase-1, ecto-5′-NT, and eADA enzymes in the murine vessels of the cerebral cortex was studied using the immunofluorescent labeling. A double staining of three above-mentioned ecto-enzymes with CD31, being a constitutive marker of the vascular endothelium, was performed. Coronary sections of the brain were rinsed in PBS, then incubated in blocking solution (5% normal goat serum (NGS)/0.3% Triton X-100 in PBS) for 30 min at RT. Subsequently, the sections were incubated in solution with primary antibodies: rabbit polyclonal CD39/ENTPD1 (Proteintech, USA; 1:200), rabbit polyclonal 5′-Nucleotidase/CD73 (NOVUSBIO, USA; 1:3000), rabbit polyclonal adenosine deaminase/ADA (NOVUSBIO, USA; 1:7000), mouse monoclonal CD31/PECAM-1 (NOVUSBIO, USA; 1:100) for 24 h at 4 °C. The sections were washed in PBS and incubated with the secondary antibody: goat anti-mouse conjugated to Alexa Fluor 488 (Thermo Fisher Scientific Inc., USA; 1:300) and goat anti-rabbit conjugated to Cy3 (Invitrogen, USA; 1:500) for 2.5 h at RT. The sections were washed in PBS, then mounted on slides, dried, and coated with Kaiser's gelatin (Sigma, UK). To verify the specificity of the immunohistochemical staining, the omission tests were done by omitting the primary or secondary antibody, respectively. Trials using a solution of Protein Block were also performed to exclude non-specific binding (BioGenex, CA). For the immunohistochemical assessment of the ecto-enzymes, the cortical brain vessels originating from the frontal and parietal regions were chosen. Verification of the cortical areas was done on the basis of the Mouse Brain in Stereotaxic Coordinates (Paxinos and Franklin 2019).

### Microscopy Imaging

A confocal laser scanning microscopy system LSM 880 (Zeiss, Germany) mounted on a microscope AxioImager.Z2 (Zeiss, Germany) was used in this study. Images were obtained with a 20 × objective lens with a zoom of 1.0 times. Verification of the immunofluorescent sections in the non-corresponding channels was done in order to eliminate the spectral bleed-through. The analysis of the obtained images was done using the image analysis program Zen 2.3 (Blue Edition; Zeiss). Using the colocalization tools in ZEN 2.3 program, the occurrence and intensity of colocalizations of selected combinations of fluorescent markers used in the study were assessed. The intensity of pixel signals for two channels representing fluorescent markers is presented on *X* and *Y* axes of the plot. The intensity of pixel signals increases distally on both axes. The scatter plot represents all the pixels of the image organized by intensity. Pixels representing double staining (colocalization) are located in the central part of the scatter plot area. At the same time, double-stained (colocalizing) pixels of higher signal intensities occupy the central and right-upper part of the scatter plot area.

Additionally, in order to confirm the colocalization, the signal intensity was analyzed for individual channels, representing specific fluorescent markers, in the selected cross-sectional planes of the chosen structures. The signal intensity for each channel is presented as linear plot. An increase in signal intensity for both channels occurring at the same point on the linear plot indicates the colocalization.

### Statistical Analysis

All statistical analyses were performed using the statistical Scientific Graphing Software GraphPad Prism v 5.0.3 (GraphPad Software, Inc., CA). The normal distribution of values was verified by the Shapiro–Wilk test. Student’s t-test was used for the analysis of data characterized by a normal distribution of values. The Mann–Whitney non-parametric test was used for the analysis of the values which did not demonstrate normal distribution. Differences between experimental groups were considered significant at *p* < 0.05. Data were presented as mean ± standard error of the mean (SEM).

## Results

### Hypercholesterolemia Does Not Induce Changes in Intracellular Concentration of ATP and NAD in mBMECs

**Fig. 2:**
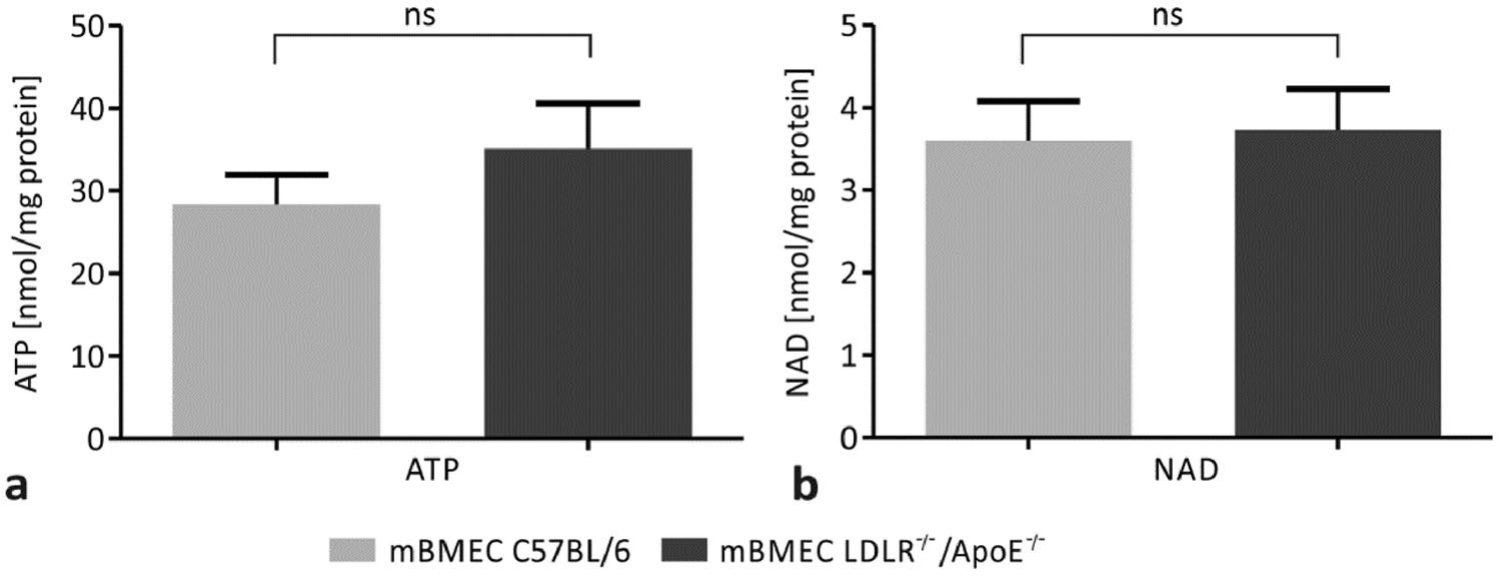
Effect of hypercholesterolemia on the intracellular concentrations of **a** ATP and **b** NAD in C57BL/6 and LDLR^−/−^/Apo E^−/−^ murine brain microvascular endothelial cells (mBMECs). No significant differences in the intracellular concentrations of ATP and NAD between mBMECs of LDLR^−/−^/Apo E^−/−^ experimental mice and C57BL/6 control mice were detected. The Mann–Whitney test was used for the comparison of ATP concentrations. Student’s t-test was used for the comparison of NAD concentrations between LDLR^−/−^/Apo E^−/−^ mice and control mice. Data are presented as mean ± SEM

In order to determine the effect of hypercholesterolemia on purinergic nucleotides, the intracellular concentrations of ATP and NAD were studied in mBMECs of LDLR^−/−^/Apo E^−/−^ mice and C57BL/6 control mice. The mean value of ATP concentration in mBMECs of the experimental group was 35.1 ± 5.4 nmol/mg protein, whereas in C57BL/6 mice it was 28.4 ± 3.6 nmol/mg protein (Fig. 2a). Results showed no significant difference in ATP concentration between the two groups (*p* = 0.6673). The concentration of NAD in mBMECs of LDLR^−/−^/Apo E^−/−^ mice was 3.7 ± 0.5 nmol/mg protein, whereas in C57BL/6 mice it was 3.6 ± 0.5 nmol/mg protein (Fig. 2b). We did not detect significant differences in NAD concentration between experimental and control mice (*p* = 0.8527). Reassuming, our results show no hypercholesterolemia-induced changes in the concentration of ATP and NAD in mBMECs.

### Hypercholesterolemia Does Not Affect the Glycolytic Function of mBMECs

**Fig. 3:**
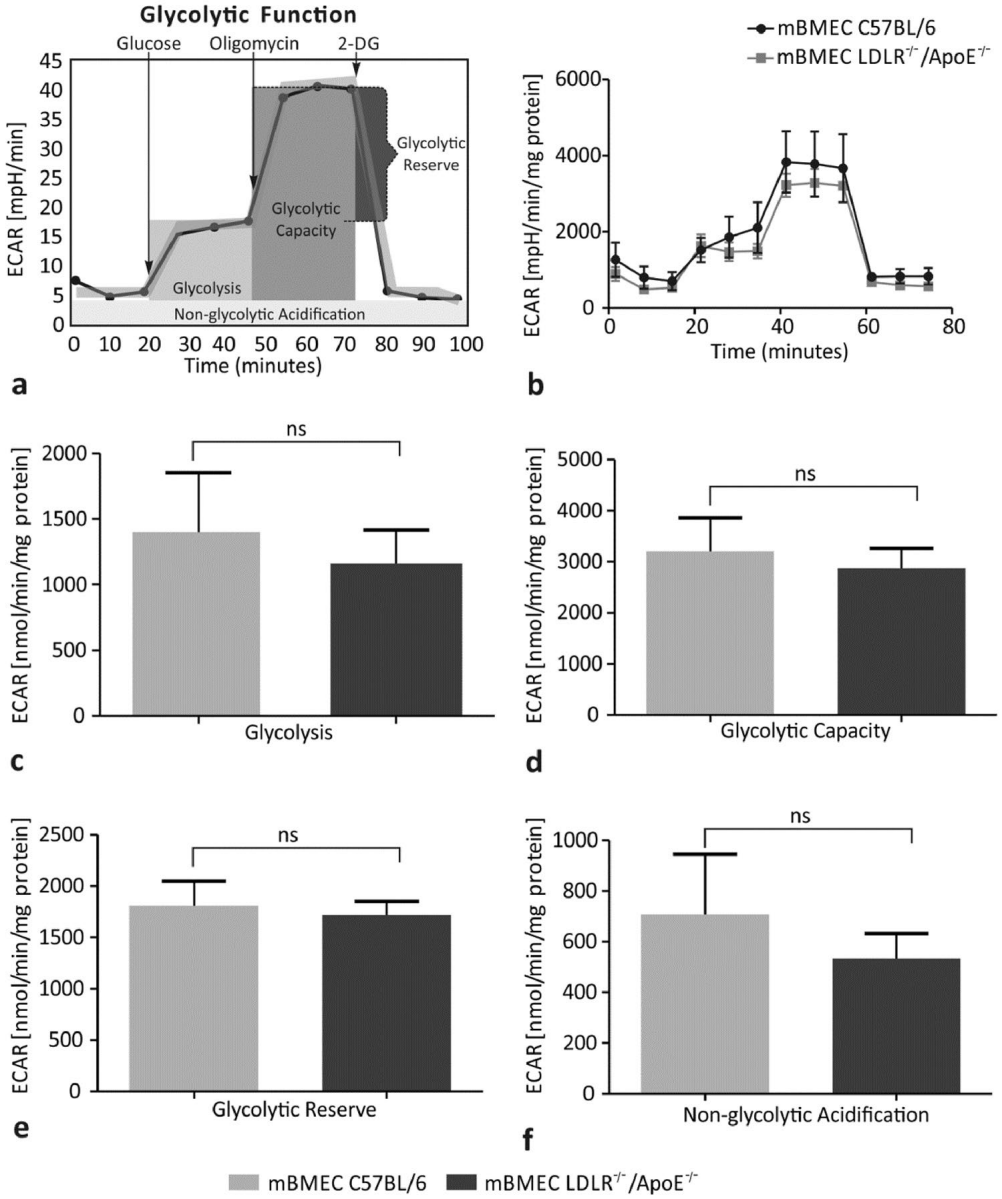
Effect of hypercholesterolemia on parameters of the glycolytic function: **a** test profile, **b** the extracellular acidification rate (ECAR), **c** glycolysis, **d** glycolytic capacity, **e** glycolytic reserve, and **f** non-glycolytic acidification in the murine brain microvascular endothelial cells (mBMECs) from C57BL/6 and LDLR^−/−^/Apo E^−/−^ mice. Glycolytic capacity, glycolytic reserve, and non-glycolytic acidification did not show a significant difference between the experimental and control groups. Mann–Whitney test was used to compare the studied parameters between hypercholesterolemic mice and control mice. Data are presented as mean ± SEM

The glycolytic function was analyzed based on ECAR in experimental LDLR^−/−^/Apo E^−/−^ mBMECs and control C57BL/6 mBMECs (Fig. 3a, b). The glycolysis value in the experimental group was 1159 ± 257 mpH/min/mg protein, whereas in the control group-1398 ± 455 mpH/min/mg protein (Fig. 3c). No significant difference between these values is reported (*p* = 0.3154). The glycolytic capacity value in the experimental group was 2877 ± 382 mpH/min/mg protein, whereas in the control group-3202 ± 659 mpH/min/mg protein (Fig. 3d). No significant difference between these values is reported (*p* = 0.6038). The glycolytic reserve values in experimental and control groups were 1718 ± 133 mpH/min/mg protein and 1804 ± 242 mpH/min/mg protein, respectively, and no significant difference between them is reported (*p* = 0.4967; Fig. 3e). The non-glycolytic acidification value was 532 ± 99 mpH/min/mg protein in the experimental group and 707 ± 236 mpH/min/mg protein in the control group (Fig. 3f). No significant difference between these two values is present (*p* = 0.9048). Altogether, analysis of the glycolytic function measured as the ECAR in mBMECs did not show significant differences between the experimental and control mice.

### Hypercholesterolemia Increases the Extracellular Activity of Ecto-5′-NT and eADA in mBMECs

**Fig. 4:**
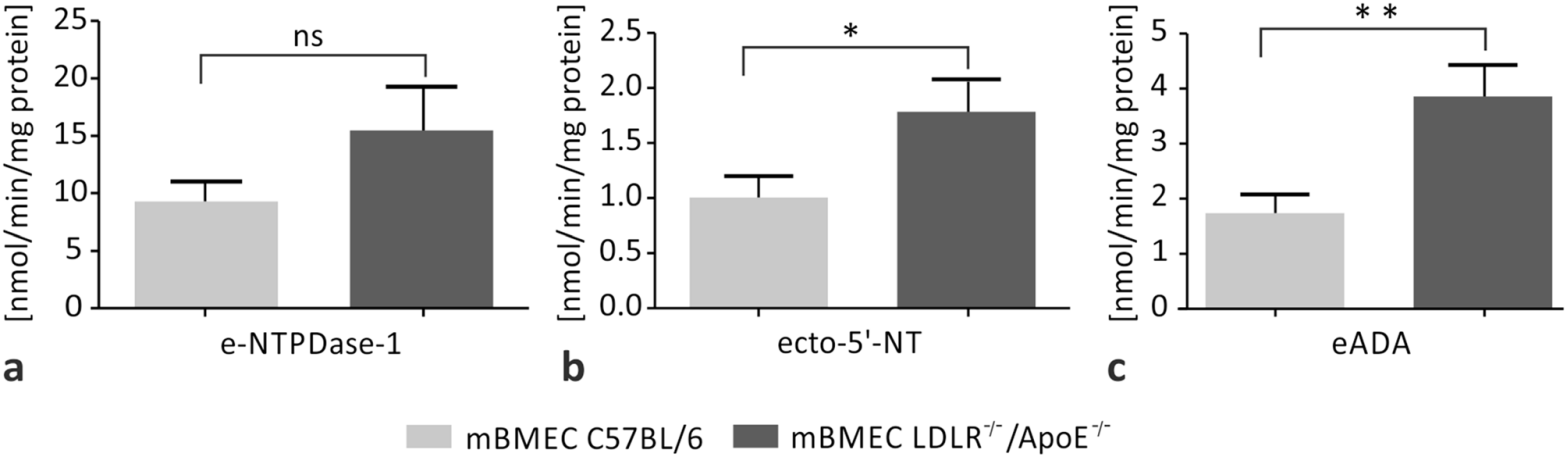
Effect of hypercholesterolemia on the extracellular activity of **a** ecto-nucleoside triphosphate diphosphohydrolase-1 (e-NTPDase-1), **b** ecto-5′-nucleotidase (ecto-5′-NT), and **c** ecto-adenosine deaminase (eADA) in mBMECs of C57/BL6 control group mice and LDLR^−/−^/Apo E^−/−^ experimental mice. The activities of extracellular ecto-5′-NT and eADA enzymes are higher in mBMECs of LDLR^−/−^/Apo E^−/−^ mice than in the C57/BL6 group. Mann–Whitney test was used to compare the enzyme activity between hypercholesterolemic and control mice. Data are presented as mean ± SEM; *p < 0.05, **p < 0.01

The extracellular activity of three ecto-enzymes: e-NTPDase-1/CD39, ecto-5′-NT/CD73, and eADA was measured in mBMECs of LDLR^−/−^/Apo E^−/−^ and C57/BL6 wild-type mice. The values of the extracellular activity of e-NTPDase-1 in the experimental and control group were 15.5 ± 3.8 mpH/min/mg protein and 9.3 ± 1.7 mpH/min/mg protein, respectively, and they did not differ significantly between the groups (*p* = 0.1581; Fig. 4a). The extracellular activity of the ecto-5′-NT in the experimental and control groups were 1.8 ± 0.3 mpH/min/mg protein and 1.0 ± 0.2 mpH/min/mg protein, respectively. Results showed a significant difference between the two groups (*p* = 0.0439; Fig. 4b). The extracellular activity of eADA was 3.8 ± 0.6 mpH/min/mg protein in the hypercholesterolemic group and 1.7 ± 0.3 mpH/min/mg protein in the control group. Statistical analysis showed a difference between these groups (*p* = 0.0078; Fig. 4c). In summary, hypercholesterolemia induces an increase in the extracellular activity of ecto-5′-NT and eADA in mBMECs.

### Hypercholesterolemia Does Not Affect the Localization and Immunoreactivity of the Ecto-enzymes in mBMECs

**Fig. 5:**
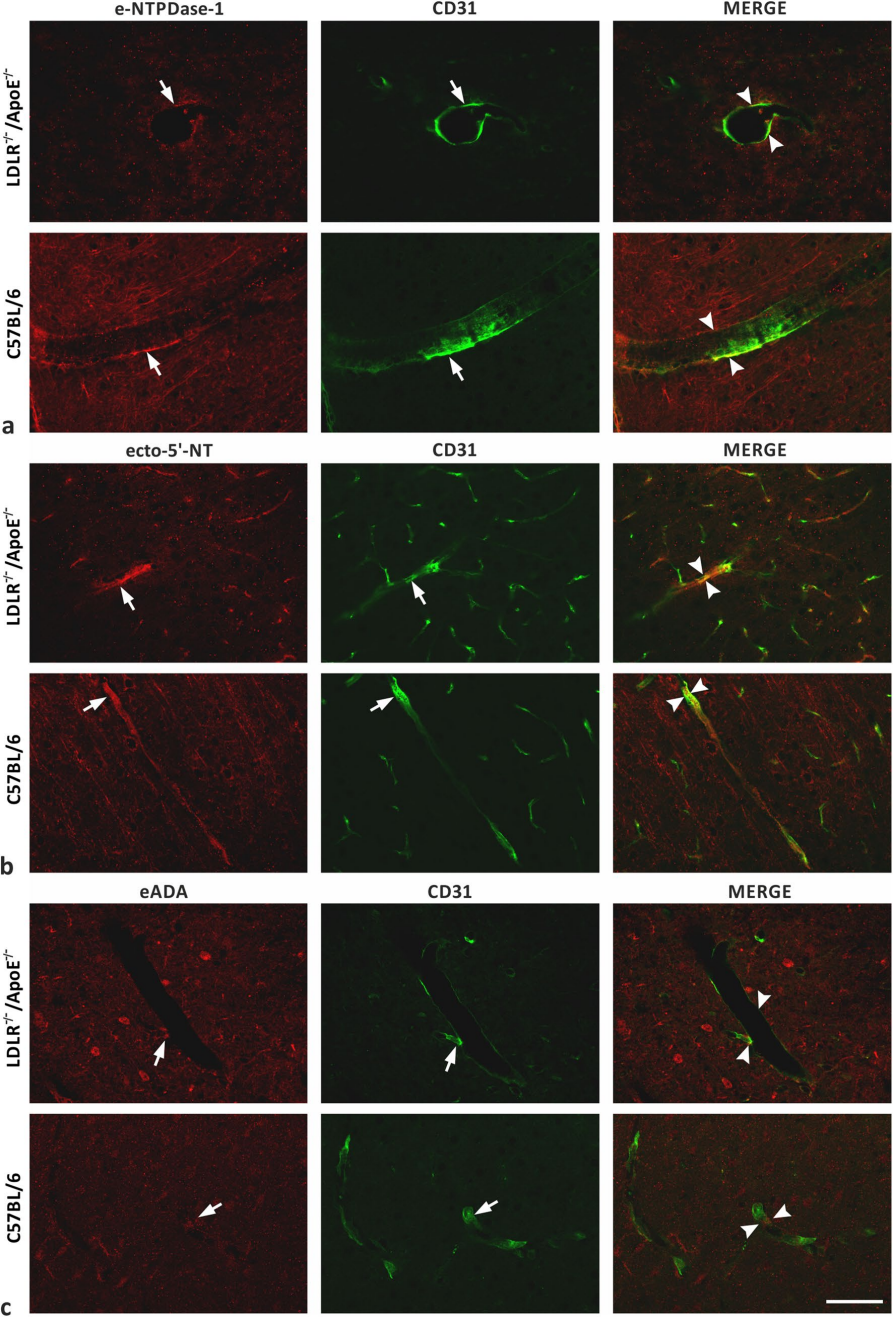
Double-staining of **a** e-NTPDase-1 with CD31, **b** ecto-5′-NT with CD31, and **c** eADA with CD31 in cortical cerebral blood vessels of 3-month-old LDLR^−/−^/Apo E^−/−^ experimental and C57/BL6 control group mice. Ecto-enzymes immunoreactivity in CD31-positive endothelial cells is indicated with the arrows. Arrowheads were used to mark the location of the line for colocalization and signal strength analysis. No apparent differences in localization and immunoreactivity of all studied ecto-enzymes in endothelial cells between LDLR^−/−^/Apo E^−/−^ and C57/BL6 mice are observed. Scale bar = 50 μm

In order to verify the presence of the ecto-enzymes e-NTPDase-1, ecto-5′-NT, and eADA in the murine cortical brain vessels, double staining with CD31, a marker of the endothelial cells, was performed (Fig. 5a–c). In both the hypercholesterolemic and control group, all three ecto-enzymes were present in the endothelial cells. There was no apparent difference in the location of immunoreactivity between these two groups.

**Fig. 6:**
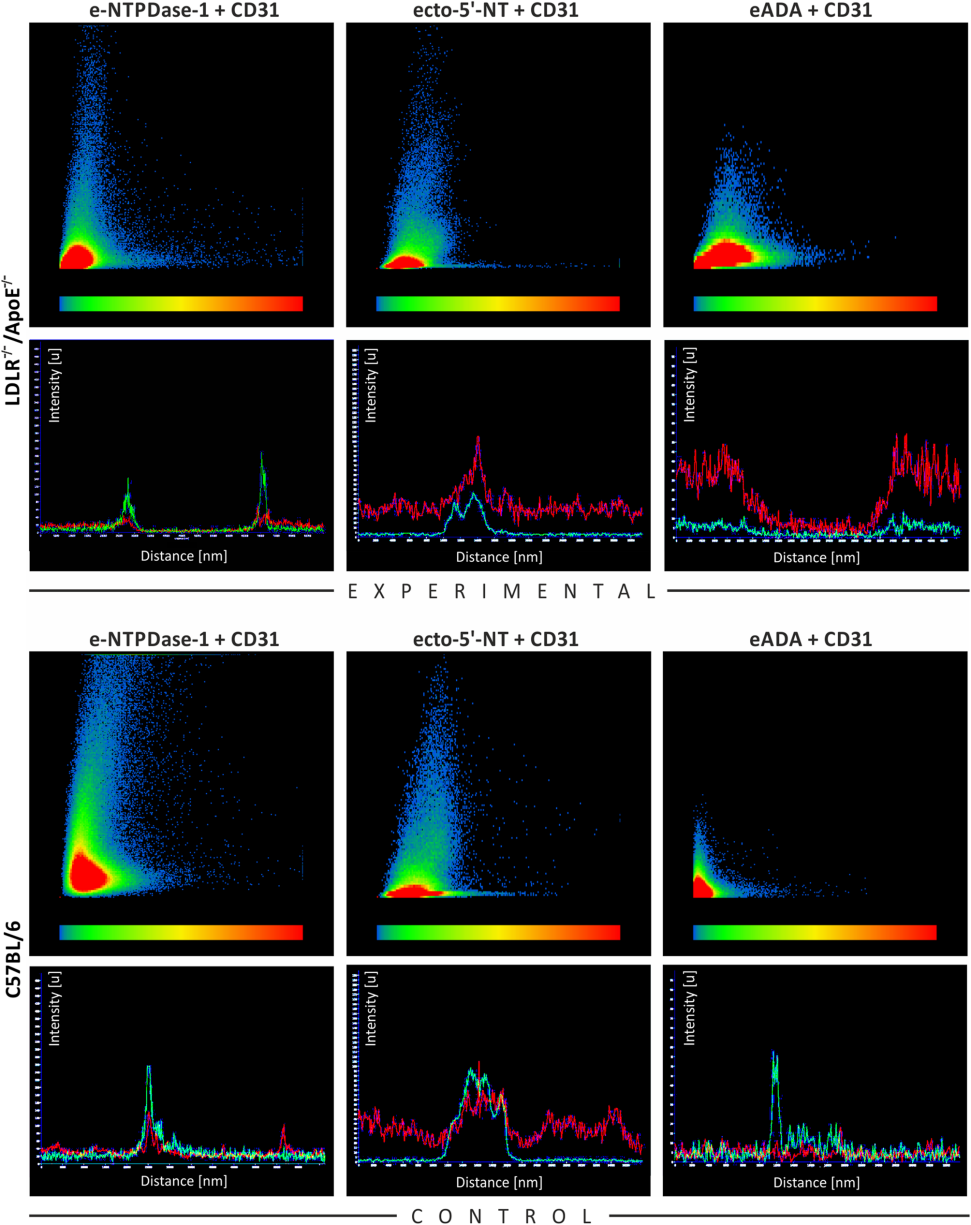
The analysis of colocalization and signal intensity of double stainings for e-NTPDase-1 + CD31, ecto-5′-NT + CD31, and eADA + CD31. The intensities of pixel signals for two channels representing fluorescent markers are presented on *X* and *Y* axes in arbitrary units. Pixels along the *X*-axis (for CD31 staining) and along the *Y*-axis (for e-NTPDase-1, ecto-5′-NT, and eADA staining) represent the single pixel signals. The intensity of pixel signals increases distally on both axes. The colocalizing pixels representing overlap of channels are situated in the central part of the scatter plot area. Additionally, the signal intensities for individual channels in selected cross-sectional planes of the chosen structures are presented in linear plots. An increase in signal intensity for both channels occurring at the same point on the linear plot indicates the colocalization. The intense colocalizations of e-NTPDase-1 and ecto-5′-NT with CD31 are found in endothelial cells in EX and WT mice, whereas the lower intensity of eADA colocalization with the CD31 in both EX and WT mice is reported

The occurrence and intensity of colocalizations of e-NTPDase-1 with CD31, ecto-5′-NT with CD31, as well as eADA with CD31 in endothelial cells were verified by means of the colocalization tools in both EX and WT mice (Fig. 6). The colocalizations were confirmed. Pixels representing double staining are located in the central parts of the scatter plot. Furthermore, the increases in signal intensity for channels representing e-NTPDase-1 and CD31, as well as ecto-5′-NT and CD31, occurring at the same points on the linear plots indicate the colocalizations. Considering the fact that eADA is found not only in the endothelial cells but also in astrocytes and neurons, its colocalization with the CD31 although present, is not as intense, as it is in the case of the above-mentioned enzymes, in both EX and WT mice.

## Discussion

In this study, we aimed at determining the parameters of energy metabolism on the basis of changes in ATP and NAD and glycolytic activity in the mBMECs. We also studied the effect of hypercholesterolemia on the activity of ecto-enzymes (e-NTPDase-1, ecto-5′-NT, and eADA), which are crucial for the purinergic metabolism in these cells. While no changes in the parameters of energy metabolism, such as ATP and NAD or the glycolytic function were found in these cells, the results showed higher activity of two ecto-enzymes: ecto-5′-NT and eADA in hypercholesterolemic mice, compared with the control group.

The inability to refer to the previously published results, even taking into account the autonomy of cholesterol metabolism in the brain (see Czuba et al. 2017), made us create a working hypothesis, which assumed the influence of hypercholesterolemia on the selected parameters of the energy metabolism leading to a reduction of its effectiveness. However, the obtained results negatively verified our initial assumptions.

The increased activity of the ecto-enzymes in the hypercholesterolemic mice indicates an augmented transformation of purine nucleotides in the extracellular space surrounding the vascular endothelial cells. This may be the result of changes in metabolic conditions occurring in this environment, due to the increased content of cholesterol metabolism products (e.g., oxysterols) (Czuba et al. 2017). It is also associated with the activation of the inflammatory reaction (i.e., the release of pro-inflammatory cytokines) and with disturbances in the regulation of the cerebral blood flow and blood–brain barrier leakage (Huang et al. 2021).

The relationship between hypercholesterolemia and neurodegenerative diseases has already been established (Kivipelto et al. 2002a, b; Liu et al. 2010; Czuba et al. 2017; Huang et al. 2019; Wu et al. 2022). Although the mechanisms have not been fully elucidated, there are strong premises for its existence, and hypercholesterolemia is considered a significant risk factor for neurodegenerative diseases, such as Alzheimer’s, and Parkinson’s, Niemann–Pick, and Huntington’s diseases. The mechanisms linking the dysfunction in the cholesterol metabolism with the particular neurodegenerative diseases are still the subject of intensive research (see below).

In Alzheimer’s disease, changes in the content of oxysterols, such as 24S-hydroxycholesterol and 27-hydroxycholesterol, occurring in the course of hypercholesterolemia correlate with cognitive decline (Liu et al. 2010; Wu et al. 2022). Hypercholesterolemia increases the production and accumulation of amyloid beta (Aβ) in the brain, hyperphosphorylation of Tau protein, and ultimately leads to neuronal death (Ghribi et al. 2006b). Astrocyte activation also links hypercholesterolemia with the development of Alzheimer’s disease (Chen et al. 2016). It results in the release of pro-inflammatory cytokines (e.g., IL-1β), as well as an increased expression of apolipoprotein E (APOE) and aquaporin 4 (AQP4), associated with cholesterol transport (Czuba et al. 2017). A correlation between the APOE genotype and Aβ removing ability from the perivascular space has also been found and the APOE ε4 allele expression favoring Aβ accumulation has been reported (Hawkes et al. 2015). In addition to the accumulation of Aβ, the release of inflammatory factors and oxidative stress are associated with disturbances in energy metabolism. All these factors enhance neurodegenerative changes (Chen et al. 2016; Czuba et al. 2017).

In Parkinson’s disease, increased aggregation of α-synuclein is responsible for damaging of the dopaminergic neurons in the substantia nigra (Liu et al. 2010). In these cells, an increased content of cholesterol metabolites such as 24- and 27-hydroxycholesterols in the course of hypercholesterolemia was found and their presence affected the amount of α-synuclein accumulation. Although the explanation of the direct mechanism of α-synuclein damaging effect on the brain tissue requires further research, it can be assumed that it coexists with the inflammatory reaction and oxidative stress. All these processes lead to a decrease in the efficiency of energy metabolism and cell death (Liu et al. 2010).

In Niemann–Pick disease, an accumulation of cholesterol and sphingolipid in endosomes and lysosomes of neurons in many brain areas was reported, which is a result of lipid transport impairment (Liu et al. 2010). Further research is needed to explain the direct neurodestructive effect of cholesterol and its role in the endothelial cells contributing to pathological processes in this disease.

A link between hypercholesterolemia and neurodegeneration, as well as changes in adenine nucleotide metabolism have also been reported in the cellular model of Huntington’s disease (Toczek et al. 2018). Toczek et al. showed reduced intracellular concentration of ATP and reduced activities of ecto-5′-nucleotidase (esto-5'-NT), ecto-nucleoside triphosphate diphosphohydrolase (eNTPDase-1), and ecto-adenosine deaminase (eADA). However, the NAD^+^ concentration remained unchanged. These changes indicate a possible reduction of intracellular energy transformations and a simultaneous reduction of extracellular nucleotide breakdown.

Summarizing these data, the relationship between the increase in cholesterol content and the severity of neurodegeneration was reported in many pathophysiological processes. However, not in all cases it has been possible to explain the direct mechanisms of cholesterol or its intermediate metabolites action upon nervous tissue and, in particular, on the vascular system. Further research, which involves the assessment of the extent of energy metabolism impairment, is warranted. It can be assumed that the size of this impairment varies among the diseases, depending on the type and the extent of damage to the constituents of the neurovascular unit.

Induced by hypercholesterolemia, high activity of ecto-enzymes leads, in turn, to an accelerated adenosine and inosine turnover in the extracellular space. Changes in the proportion between ATP, which has pro-inflammatory and pro-apoptotic properties, and adenosine and inosine, which have anti-inflammatory and neuroprotective properties, may significantly influence processes in the extracellular space of the mBMEC (Wang et al. 2023). This interpretation is supported by the increased activity of ecto-5′-NT and eADA, responsible for the conversion of AMP to adenosine and adenosine to inosine, respectively. Further research is needed to explain the significance of these processes. This may be important both for understanding the regulatory mechanisms in the CNS involving the purinergic system and for developing effective therapeutic strategies to limit the impact of the impaired lipid metabolism on the cerebrovascular system.

The relationship between changes in the activity of ecto-enzymes in vascular endothelial cells and hypercholesterolemia followed by atherosclerosis was observed in animal models such as ApoE^−/−^LDLR^−/−^ double knockout mice (Kutryb-Zajac et al. 2014, 2016; Zukowska et al. 2015). A close relationship has been demonstrated between changes in the concentration of ecto-enzymes and serum lipid profile parameters and the degree of activity of endothelial cells (Kutryb-Zajac et al. 2020a). Whereas the ecto-5′-nucleotidase activity was moderately decreased, the activity of eADA increased significantly in endothelial cells of the atherosclerotic vessels and activated macrophages. The changes in the activities of the ecto-enzymes correspond to decreased production and increased degradation of the extracellular adenosine (Kutryb-Zajac et al. 2018a). The correlation between these changes and the progression of atherosclerosis also has been reported. The postulated mechanism of purinergic nucleotides and adenosine effect on atheromatosis is based on the regulation of the inflammatory and thrombotic processes. The mechanisms responsible for initiating changes in the activity of ecto-enzymes and energy metabolism parameters, induced by hypercholesterolemia, include the immunological reaction, infiltration of activated macrophages and microglia, as well as oxidative stress and hypoxia (Zukowska et al. 2015; Kutryb-Zajac et al. 2018a).

It should be emphasized, however, that the extent of the hypercholesterolemia-induced energy metabolism impairment varies and depends on the conditions present in a specific experimental model, as well as coexisting factors. Studies on the effect of factors such as uric acid, glucose, atorvastatin, acetylsalicylic acid, monounsaturated and polyunsaturated fatty acids, as well as IL-6 on murine-immortalized heart endothelial cells did not show their effect on changes of the intracellular ATP and ADP concentrations (Kutryb-Zajac et al. 2018b). Only an increase in the intracellular concentration of NAD^+^ was reported, after the use of polyunsaturated fatty acids and atorvastatin. This indicates rather a moderate effect of the tested substances on the energy metabolism in the murine endothelial cells. These observations are mostly in line with our results concerning changes in the energy metabolism in the vascular endothelial cells of the cerebral vessels in the hypercholesterolemic mice.

Previous studies have shown a higher rate of eADA-catalyzed conversion of adenosine to inosine in the atherosclerotic wall of aortoiliac bifurcation in LDLR^−/−^/ApoE^−/−^ mice (Kutryb-Zajac et al. 2014). This suggests a decrease in the availability of adenosine in the arterial wall in the course of atherosclerosis. In addition, the increased eADA activity has been found in many diseases of the vascular system, including atherosclerosis, hypertension, and myocardial ischemia, summarized in Kutryb-Zajac et al. (2020b). The increase in the eADA activity observed in our study, combined with the postulated decrease in adenosine content, may indicate the similarity of pathomechanisms occurring in many parts of the vascular system in the course of cardiovascular diseases. Therefore, modulation of the activity of this enzyme could have a potential therapeutic value.

Our results did not show the effect of hypercholesterolemia on the glycolytic parameters in mBMECs. This can be explained in two ways: either hypercholesterolemia has no effect on the level of glucose metabolism in the studied cell population, or hypercholesterolemia lasted too short and had too little effect on glycolysis in the experimental model used. Further research is needed to clarify this issue.

Interestingly, our results showed no changes in ATP and NAD content between the control and experimental group. This correlated to the lack of changes in the studied glycolytic parameters. These results can be interpreted as a reflection of the stable conditions of energy metabolism occurring in the hypercholesterolemic model used. The constant concentration of nucleotides can be achieved by induction of DAMPs, also known as alarmins (Di Virgilio et al. 2009; Walko et al. 2014). DAMPs include both nuclear and cytoplasmic proteins (e.g., heat shock proteins, S100 proteins, histones, and interleukin-1α) present in the microglia and astrocytes. They are released from the cells into the extracellular milieu after tissue damage or stress and ultimately activate the innate immune system (Di Virgilio et al. 2009).

In addition, unchanged ATP concentration may result from the inhibition of the ATP release pathway e.g., based on the mechanism mediated by the vesicular nucleotide transporter (VNUT) expressed by endothelial cells (Hasuzawa et al. 2020). VNUT is responsible for the loading and storage of ATP in the intracellular secretory vesicles (Lim To et al. 2015). It can be speculated that this system, present in endothelial cells, is negatively affected by elevated levels of cholesterol and its metabolites. Hypothetically, the increased activity of extracellular enzymes: ecto-5′-NT and eADA, with an unchanged intracellular concentration of ATP and NAD, could be the result of impaired cell membrane permeability. In this case, the endothelial cells would maintain a constant concentration of purine nucleotides to limit changes in their metabolism. At the same time, maintaining the appropriate concentration of ATP, adenosine, and inosine in the extracellular space would require the increased activity of the ecto-enzymes.

## Conclusions

Our results show the hypercholesterolemia-induced changes in the ecto-enzyme-regulated extracellular purine nucleotide transformations, which activate both extracellular adenosine production and adenosine conversion to inosine pathways in the cerebral microvascular endothelial cells. We did not observe alterations in energy equilibrium that could limit the damage caused by the increased levels of cholesterol and its metabolites. Our results may be a starting point for new research and an inspiration for designing new therapeutic strategies based on the regulation of the activity of the ecto-enzymes responsible for the transformation of purine nucleotides.

## Data Availability

The datasets generated during and/or analyzed during the current study are not publicly available because they do not infringe the individual's privacy but are available from the corresponding author on reasonable request.

## Abbreviations

Aβ: β-Amyloid
APOE: Apolipoprotein E
AQP4: Aquaporin 4
BBB: Blood-brain barrier
CNS: Central nervous system
eADA: Ecto-adenosine deaminase
DAMP: Deoxyadenosine monophosphate
DMEM: Dulbecco’s Modified Eagle’s Medium
ECAR: Extracellular acidification rate
ecto-5′-NT: Ecto-5′-nucleotidase
EHNA: Erythro-9-(2-hydroxy-3-nonyl) adenine
e-NTPDase: e-NTPDase-1: Ecto-nucleoside triphosphate diphosphohydrolase
FBS: Fetal Bovine Serum
HBSS: Hanks' Balanced Salt solution
HEPES: N-2-hydroxyethylpiperazine-N-2-ethane sulfonic acid
HPLC: High-performance liquid chromatography
mBMECs: Murine brain microvascular endothelial cells
NAD: Nicotinamide adenine dinucleotide
P1R: P1 purinoreceptors
P2XR: P2X purinoreceptors
P2YR: P2Y purinoreceptors
VNUT: Vesicular nucleotide transporter
